# Supplementary material for: Gene Expression Pattern of ESPL1, PTTG1 and PTTG1IP Can Potentially Predict Response to TKI First-Line Treatment of Patients with Newly Diagnosed CML
Source: Cancers (Basel). 2023 May 8;15(9):2652. doi: 10.3390/cancers15092652 (PMC10177117; doi:10.3390/cancers15092652)
Supplement: Supplementary file 1 [file cancers-15-02652-s001.zip › Table S1.pdf]

CML patients characteristics

No.	Time until MMR (M)	*BCR::ABL1* oncogene fusion type variants	Sex	Age (a)	Leukocytes/ul	*BCR::ABL1* quotient (%)	Q(IS)	Initial therapy	*ESPL1* (ΔC$_t$ value)	*PTTG1* (ΔC$_t$ value)	*PTTG1IP* (ΔC$_t$ value)	Distance
NR (n=51)												
NR-1	> 12	e13a2	m	57	4800	18	-	Nilotinib	5.82	3.66	2.40	1.1509645
NR-2	> 12	e13a2	m	49	97 300	76	-	Nilotinib	4.56	4.04	6.06	3.48104761
NR-3	> 12	e13a2	m	45	21 800	22	-	Dasatinib	4.65	3.74	1.44	1.24452253
NR-4	> 12	e14a2	m	63	399 000	100	-	Imatinib, bosutinib	3.38	3.70	1.48	1.79366908
NR-5	14	e13a2, e14a2	f	29	81 400	31	-	Nilotinib + IFNα	4.28	3.81	4.00	1.48897486
NR-6	14	e13a2, e14a2	f	48	36 700	61	-	Imatinib	2.95	2.55	1.96	2.08587427
NR-7	> 14	e13a2, e14a2	f	67	34 000	14	-	Imatinib	2.94	2.77	4.22	2.46980874
NR-8	15	e14a2	f	61	5800	13	-	Imatinib	6.44	3.86	-0.61	3.69841823
NR-9	16	e14a2	m	83	2600	0.62	0.48	Imatinib	6.65	3.49	1.96	2.04215268
NR-10	16	e14a2	f	41	8000	0.15	0.13	Nilotinib, + IFNα	8.26	8.69	10.29	9.94418452
NR-11	16	e14a2	m	47	4500	31 *	-	Imatinib, dasatinib	5.64	5.76	2.67	2.51663932
NR-12	> 16	e14a2	m	81	3500	25	-	Imatinib + IFNα	7.38	3.29	1.19	3.02848383
NR-13	17	e13a2, e14a2	m	75	40 200	52	-	Dasatinib	3.99	3.13	4.54	2.05719732
NR-14	17	e13a2	m	51	6500	0.69	0.53	Nilotinib	6.15	3.73	-0.86	3.78911643
NR-15	> 17	e13a2	m	59	75 500	51	-	HU, imatinib	2.84	2.73	1.38	2.36368105
NR-16	18	e13a2	m	27	168 400	131	-	Imatinib,dasatinib	5.37	5.06	2.93	1.79321232
NR-17	18	e13a2	m	41	18 200	52 *	-	SZT §	3.62	2.61	1.24	1.95028029
NR-18	> 18	e14a2	m	57	57 000	33	-	Nilotinib, + IFNα	3.36	0.51	2.61	3.20528788
NR-19	19	e13a2	f	66	256 150	43 *	-	Imatinib	2.32	1.81	1.36	3.15674694
NR-20	19	e13a2	f	68	80 000	4.6 *	4.0 *	Imatinib	3.69	3.76	2.19	1.17718998
NR-21	> 20	e1a2	m	87	52 300	38	-	HU, nilotinib, + IFNα	3.53	3.26	1.50	1.65285429
NR-22	21	e13a2, e14a2	f	33	9100	17	-	Nilotinib	7.95	7.28	9.65	8.63501389
NR-23	21	e14a2	m	40	404 000	80	-	Nilotinib	2.66	1.95	3.30	2.61145556
NR-24	21	e14a2	f	58	2000	0,99	0.76	Nilotinib, imatinib	5.75	3.23	-0.79	3.57913382
NR-25	21	e14a2	m	49	4900 *	2.3	1.8	Nilotinib	2.76	1.78	1.34	2.86096701
NR-26	> 21	e14a2	f	67	23 900	52	-	HU, nilotinib	2.57	2.03	1.68	2.73141038
NR-27	23	e13a2	m	49	61 400	26	-	HU, nilotinib, imatinib	7.40	8.42	11.21	10.2821235
NR-28	23	e14a2	f	70	297 400	7.1 *	6.2 *	Imatinib	1.57	1.29	2.54	3.80218629
NR-29	23	e13a2, e14a2	m	76	40 400	28 *	-	Imatinib	5.02	1.54	-0.11	3.33436272
NR-30	25	e13a2, e14a2	f	48	113 700	12 *	-	Imatinib	2.83	4.48	2.88	2.18959133
NR-31	25	e14a2	f	70	80 100	9.6 *	8.4 *	Imatinib	3.61	2.48	2.14	1.53416077
NR-32	26	e14a2	m	50	118 000	66	-	Nilotinib + IFNα, dasatinib	4.39	3.72	4.68	2.096926
NR-33	27	e13a2	m	50	149 700	4.4 *	3.9 *	Imatinib	3.48	2.57	3.45	1.70805384
NR-34	28	e14a2	m	59	9300	20 *	-	Imatinib §	8.33	0.69	6.46	5.91799879
NR-35	29	e13a2, e14a2	m	78	5200	2.1	1.8	Imatinib	6.79	3.24	1.31	2.46586457
NR-36	29	e13a2	m	28	380 000	39	-	Ara-C, dasatinib, nilotinib	4.20	5.12	7.07	4.78144163
NR-37	29	e13a2	m	57	200 000	77	-	Imatinib	3.56	4.65	3.35	1.84053346
NR-38	30	e14a2	f	83	33 700	178	-	Imatinib	3.56	4.57	0.91	2.3763382
NR-39	31	e13a2	m	84	5300	15	-	Imatinib §	4.03	3.47	1.47	1.35525053
NR-40	33	e14a2	m	26	56 800	4.7	4.1	HU, nilotinib, + IFNα	3.18	3.15	5.22	3.02180282

NR-41	> 32	e13a2	f	60	355 000	30 *	-	Nilotinib	2.89	1.62	1.57	2.77144409
NR-42	35 †	e13a2	m	86	56 300	2.9 *	2.6 *	Imatinib	3.44	2.77	3.72	1.79973951
NR-43	36	e14a2	f	56	181 000	52	-	HU, nilotinib, + IFNα	3.50	3.03	1.62	1.63749564
NR-44	40	e13a2	f	69	59 200	102	-	HU, nilotinib, imatinib	5.05	4.13	3.22	0.98035671
NR-45	41	e14a2	f	52	87 600	43	-	Nilotinib	3.38	1.95	2.16	2.03974546
NR-46	> 42	e13a2	f	71	7200	57	-	Imatinib	4.33	3.21	1.10	1.60182682
NR-47	51	e13a2	f	49	5000	1.2	0.92	Nilotinib, bosutinib,	6.05	3.93	-0.07	3.06143084
NR-48	51	e14a2	f	57	264 000	45	-	Imatinib	2.69	2.11	5.34	3.62448904
NR-49	64	e13a2	m	67	45 000	20 *	-	Imatinib	3.72	0.22	2.66	3.34715154
NR-50	86	e13a2	m	50	120 000	4.8 *	4.2 *	Imatinib §	4.10	3.14	1.49	1.33599705
NR-51	> 91	e13a2	m	82	11 000	8.3	7.3	Imatinib	10.47	6.21	1.85	6.43528565
R (n=46)												
R-1	<1	e13a2	m	56	7300	0.016 *	0.012 *	Dasatinib	6.31 #	4.27 #	0.61 #	2.710390892
R-2	1	e13a2	f	77	7000	2.8	2.5	Imatinib	6.53	3.39	1.18	2.31628687
R-3	1	e13a2	m	59	4400	0.59	0.52	Imatinib	6.60	4.53	1.09	2.67870454
R-4	2	e13a2	m	79	6900	0.40	0.35	Imatinib	6.88	4.35	2.77	2.35835513
R-5	2	e13a2	m	63	10 300	0.57	0.50	Imatinib	6.26	3.32	0.96	2.27796892
R-6	2	e13a2, e14a2	f	71	7300	30	-	Nilotinib, + IFNα	6.22	3.39	1.29	2.01502596
R-7	2	e13a2	m	85	3400	6.7	5.2	Imatinib	6.62	3.54	0.56	2.81511634
R-8	2	e13a2	m	27	3500	0.71	0.55	Nilotinib + IFNα	7.28	3.38	1.49	2.7992215
R-9	2	e14a2	f	73	7000	22	-	Nilotinib	5.47	4.09	3.89	1.60533761
R-10	2	e14a2	f	57	5800	7.8	6.0	Imatinib	5.33	3.75	2.93	0.75028429
R-11	2	e13a2, e14a2	f	65	6700	0.91	0.90	Dasatinib	6.40	3.67	0.71	2.56405509
R-12	3	e14a2	f	41	6700	1.1	0.97	HU, dasatinib	7.73	5.03	3.32	3.4813814
R-13	3	e13a2	m	28	68 600	51	-	n.k.	3.49	3.15	7.58	5.09970931
R-14	3	e14a2	m	53	12 100	1.3	1.1	Dasatinib, nilotinib	5.35	3.40	0.66	2.06936814
R-15	3	e14a2	m	84	5300	0.39	0.30	Imatinib	7.23	3.59	1.22	2.88036624
R-16	3	e13a2	f	63	19 700	68	-	Nilotinib, + IFNα	3.69	3.33	-0.76	3.54711159
R-17	3	e13a2, e14a2	m	53	208 000	48	-	Dasatinib	4.20	2.62	3.98	1.64779753
R-18	3	e13a2	m	72	6200	0.20	0.18	Nilotinib	6.69	4.99	3.11	2.56594547
R-19	4	e13a2, e14a2	f	28	178 000	78	-	Dasatinib	3.26	1.89	2.47	2.12202709
R-20	5	e13a2, e14a2	m	54	6300	0.14	0.12	Imatinib	5.66	3.45	2.46	0.95146552
R-21	5	e13a2	m	59	7100	4.8	4.2	Bosutinib	6.80	4.35	2.47	2.28305344
R-22	5	e13a2	m	28	13 800	34	-	Nilotinib	3.96	3.29	2.64	0.77309536
R-23	5	e13a2, e14a2	f	49	3500	4.2	3.2	Imatinib	6.84	3.54	0.31	3.14972273
R-24	5	e13a2, e14a2	m	48	43 800	85	-	Nilotinib	2.20	2.62	0.99	3.1151815
R-25	5	e13a2, e14a2	m	70	4300	23	-	Nilotinib	4.31	3.76	2.31	0.63311903
R-26	6	e13a2	m	14	316 000	73	-	Imatinib	2.82	3.27	1.52	2.20937021
R-27	6	e13a2, e14a2	f	54	27 600	6.8	6.0	Nilotinib	4.89	4.44	5.93	3.45230304
R-28	6	e14a2	f	70	25 600	36	-	Nilotinib	3.09	2.52	2.59	1.86434459
R-29	6	e14a2	m	85	86 600	49	-	Nilotinib, + IFNα	3.49	2.52	1.07	2.18067486
R-30	6	e14a2	m	73	26 100	53	-	Nilotinib + IFNα	3.89	2.69	2.26	1.16230935
R-31	6	e13a2	m	47	166 000	55	-	Nilotinib	2.56	2.81	1.66	2.44476357
R-32	6	e14a2	f	67	46 600	70	-	Nilotinib + IFNα	3.10	2.50	1.78	2.05186993
R-33	6	e14a2	f	50	22 000	48	-	Nilotinib	3.64	3.24	2.27	1.15696755
R-34	7	e13a2	m	76	8800	12	9.2	Nilotinib, imatinib	4.99	3.79	0.49	2.19555536
R-35	7	e14a2	f	67	38 200	33	-	Dasatinib	3.67	3.31	4.11	1.81004372

R-36	8	e13a2	m	52	43 800	35	-	Imatinib	4.26	4.07	4.60	2.12471051
R-37	8	e13a2	m	43	132 000	79	-	HU, nilotinib + IFNα	4.17	4.49	5.22	2.85642954
R-38	8	e13a2	m	46	5100	0.90	0.69	Nilotinib	6.27	3.65	0.74	2.45640781
R-39	8	e13a2	f	38	5300	40	-	Nilotinib	5.62	3.27	1.13	1.75704563
R-40	8	e14a2	f	58	8400	34	-	Nilotinib	5.91	3.06	1.89	1.44123408
R-41	8	e13a2	m	78	35 100	0.23 *	0.18 *	Imatinib	3.72	2.37	1.91	1.61878044
R-42	10	e14a2	f	84	93 700	6.4 *	5.6 *	Imatinib, nilotinib	3.29	4.18	5.40	3.20993476
R-43	11	e13a2, e14a2	m	66	70 900	58	-	Nilotinib	3.61	2.83	4.15	1.96404214
R-44	11	e13a2	f	68	5200	0.87	0.67	Nilotinib, dasatinib	5.17	3.12	2.76	0.54959446
R-45	11	e13a2	m	32	17 100	81	-	HU, nilotinib	4.58	1.07	3.25	2.42932439
R-46	12	e14a2	f	49	103 000	68	-	Nilotinib	3.04	3.16	4.34	2.40591712
Control group (n=51)												
K-1									6.60	4.72	3.60	
K-2									5.79	4.74	2.70	
K-3									3.22	1.92	1.87	
K-4									5.15	4.37	2.48	
K-5									4.52	3.28	1.76	
K-6									5.00	4.92	2.76	
K-7									5.77	4.23	3.64	
K-8									5.89	6.62	3.01	
K-9									6.83	4.47	3.44	
K-10									6.61	3.50	3.23	
K-11									5.59	3.79	3.06	
K-12									5.84	3.89	4.61	
K-13									5.88	4.20	4.41	
K-14									6.06	4.71	5.00	
K-15									6.76	3.86	4.01	
K-16									6.74	3.83	3.54	
K-17									6.16	3.89	3.18	
K-18									6.01	3.35	2.79	
K-19									5.17	4.83	3.78	
K-20									5.58	3.97	3.68	
K-21									6.02	4.28	4.45	
K-22									5.58	5.62	2.24	
K-23									6.22	4.37	3.91	
K-24									6.26	4.76	4.15	
K-25									6.25	3.63	3.92	
K-26									5.22	4.26	3.94	
K-27									7.32	4.26	2.94	
K-28									5.25	4.15	3.98	
K-29									6.39	4.33	3.05	
K-30									5.75	4.02	3.53	
K-31									5.57	3.88	3.01	
K-32									5.61	4.04	0.06	
K-33									6.52	3.43	2.58	
K-34									5.66	3.86	3.54	
K-35									4.69	4.24	3.09	
K-36									5.92	3.71	2.71	

K-37	6.16	4.15	2.24
K-38	5.43	4.08	3.66
K-39	6.89	3.83	3.33
K-40	6.26	3.93	3.29
K-41	6.40	3.45	2.50
K-42	4.72	3.26	0.88
K-43	7.38	4.32	3.99
K-44	6.09	3.92	2.84
K-45	4.63	3.67	2.74
K-46	6.51	4.03	2.33
K-47	6.69	4.34	2.99
K-48	5.77	4.01	2.17
K-49	5.79	4.44	1.80
K-50	6.48	4.09	2.13
K-51	5.96	3.76	2.22

CML, chronic myelogenous leukemia; MMR, major molecular remission; Q, *BCR::ABL1* quotient; IS, International Scale; n, number of patients; NR, nonresponder, R, responder; n.k., not known; IFNα, Interferon-α; HU, Hydroxyurea; SZT, stem cell transplantation; Ara-C, Cytarabin.

> Further data not available

§ MMR after SZT

* Due to non-sufficient sample quality the data of the following sample was used

Due to non-available cDNA material the subsequent sample (1 month later) was used

† Death by blast crisis
