# Supplementary material for: Gene Expression Pattern of ESPL1, PTTG1 and PTTG1IP Can Potentially Predict Response to TKI First-Line Treatment of Patients with Newly Diagnosed CML
Source: Cancers (Basel). 2023 May 8;15(9):2652. doi: 10.3390/cancers15092652 (PMC10177117; doi:10.3390/cancers15092652)
Supplement: Supplementary file 1 [file cancers-15-02652-s001.zip › Table S2.pdf]

Statistical data analyses on qRT-PCR data

The MEANS Procedure

N Obs	Variable	Mean	Minimum	Maximum	Std Dev	Skewness
97	ESPL1	4.7234	1.5700	10.4670	1.7041	0.6839
	PTTG1	3.4122	0.2200	8.6870	1.3514	1.0842
	PTTG1IP	2.6362	-0.8600	11.2100	2.2009	1.5566

Group	N Obs	Variable	Mean	Minimum	Maximum	Std Dev
Nonresponder	51	ESPL1	4.5316	1.5700	10.4670	1.8573
		PTTG1	3.4099	0.2200	8.6870	1.7201
		PTTG1IP	2.8701	-0.8600	11.2100	2.5744
Responder	46	ESPL1	4.9360	2.2030	7.7270	1.5080
		PTTG1	3.4147	1.0670	5.0330	0.7803
		PTTG1IP	2.3768	-0.7570	7.5770	1.6850

Gruppe	N Obs	Variable	Skewness
Nonresponder	51	ESPL1	1.1147
		PTTG1	1.0587
		PTTG1IP	1.5158
Responder	46	ESPL1	0.0608
		PTTG1	-0.3149
		PTTG1IP	0.8980

Group	N Obs	Variable	Mean	Minimum	Maximum	Std Dev
Controls	51	ESPL1	5.892	3.217	7.383	0.757
		PTTG1	4.102	1.917	6.623	0.654
		PTTG1IP	3.073	0.063	4.997	0.927

Distances and Cut-Offs

The MEANS Procedure

N Obs	Variable	N	Mean	Median	Minimum	Maximum	Std Dev
97	Cut_off	97	2.622697	2.316287	0.549594	10.282123	1.619770
	Distance	97	2.622742	2.316333	0.549649	10.282133	1.619762
	diff	97	0.000045	0.000046	0.000009	0.000054	0.000007

Group	N Obs	Variable	N	Mean	Median	Minimum
Nonresponder	51	Cut_off	51	2.977449	2.465865	0.980357
		Distance	51	2.977492	2.465910	0.980409
		diff	51	0.000043	0.000045	0.000009
Responder	46	Cut_off	46	2.229385	2.243670	0.549594
		Distance	46	2.229432	2.243716	0.549649
		diff	46	0.000046	0.000046	0.000033

Gruppe	N Obs	Variable	Maximum	Std Dev
Nonresponder	51	Cut_off	10.282123	2.021433
		Abstand	10.282133	2.021424
		diff	0.000052	0.000009
Responder	46	Cut_off	5.099709	0.869572
		Abstand	5.099743	0.869568
		diff	0.000054	0.000004

Correlation Time-MMR and Cut-offs for all CML patients

The CORR Procedure

2 With Variables:	Cut_off	Distance
1 Variables:	Time_MMR	

Spearman Correlation Coefficients, N = 97
Prob > |r| under H0: Rho=0

	Time_MMR
Cut_off	0.09925<br>0.3335
Distance	0.09925<br>0.3335

Correlation Time-MMR and Cut-offs NONRESPONDER

The CORR Procedure

2	With Variables:	Cut_off	Distance
1	Variables:	Time_MMR	

Spearman Correlation Coefficients, N = 51
Prob > |r| under H0: Rho=0

	Time_MMR
Cut_off	0.03433<br>0.8110
Distance	0.03433<br>0.8110

Correlation Time-MMR and Cut-offs RESPONDER

The CORR Procedure

2	With Variables:	Cut_off	Distance
1	Variables:	Time_MMR	

Spearman Correlation Coefficients, N = 46
Prob > |r| under H0: Rho=0

	Time_MMR
Cut_off	-0.21594<br>0.1495
Distance	-0.21594<br>0.1495

The TTEST Procedure

Variable: Dist

Group	N	Mean	Std Dev	Std Err	Minimum	Maximum
Nonresponder	51	2.9775	2.0214	0.2831	0.9804	10.2821
Responder	46	2.2294	0.8696	0.1282	0.5496	5.0997
Diff (1-2)		0.7481	1.5839	0.3221		

Group	Method	Mean	95% CL Mean	
Nonresponder		2.9775	2.4090	3.5460
Responder		2.2294	1.9712	2.4877
Diff (1-2)	Pooled	0.7481	0.1087	1.3875
Diff (1-2)	Satterthwaite	0.7481	0.1282	1.3679

Method	Variances	DF	t Value	Pr > |t|
Pooled	Equal	95	2.32	0.0223
Satterthwaite	Unequal	69.376	2.41	0.0187

Equality of Variances

Method	Num DF	Den DF	F Value	Pr > F
Folded F	50	45	5.40	<.0001

Distances - Logistic regression

The LOGISTIC Procedure

Model Information

Data Set	WORK.K1
Response Variable	Group
Number of Response Levels	2
Model	binary logit
Optimization Technique	Fisher's scoring

Number of Observations Read	97
Number of Observations Used	97

Response Profile

Ordered Value	Group	Total Frequency
1	Nonresponder	51
2	Responder	46

Probability modeled is Gruppe='Nonresponder'.

Model Convergence Status

Convergence criterion (GCONV=1E-8) satisfied.

Model Fit Statistics

Criterion	Intercept Only	Intercept and Covariates
AIC	136.213	132.129
SC	138.787	137.278
-2 Log L	134.213	128.129

Distances - Logistic regression

The LOGISTIC Procedure

Testing Global Null Hypothesis: BETA=0

Test	Chi-Square	DF	Pr > ChiSq
Likelihood Ratio	6.0842	1	0.0136
Score	5.2123	1	0.0224
Wald	4.2712	1	0.0388

Analysis of Maximum Likelihood Estimates

Parameter	DF	Estimate	Standard Error	Wald Chi-Square	Pr > ChiSq
Intercept	1	-0.8817	0.5005	3.1032	0.0781
Dist	1	0.3911	0.1892	4.2712	0.0388

Odds Ratio Estimates

Effect	Point Estimate	95% Wald Confidence Limits	
Dist	1.479	1.020	2.143

Association of Predicted Probabilities and Observed Responses

Percent Concordant	58.7	Somers' D	0.174
Percent Discordant	41.3	Gamma	0.174
Percent Tied	0.0	Tau-a	0.088
Pairs	2346	c	0.587

ROC-Analyse DISTANCE

Obs	Distance	prob_non	prob_ responder
1	0	0.29283	0.70717
2	1	0.37975	0.62025
3	2	0.47515	0.52485
4	3	0.57239	0.42761
5	4	0.66434	0.33566
6	5	0.74532	0.25468
7	6	0.81228	0.18772
8	7	0.86483	0.13517
9	8	0.90440	0.09560
10	9	0.93328	0.06672
11	10	0.95388	0.04612
12	11	0.96834	0.03166
13	12	0.97836	0.02164

Comparison Responder - Nonresponder

The MEANS Procedure

Group	N Obs	Variable	N	Mean	Median	Minimum	Maximum
Nonresponder	51	Time_MMR	51	27.324	21.500	12.500	91.500
		Age	51	58.353	57.000	26.000	87.000
		Leukocytes	51	90977.451	52300.000	2000.000	404000.000
		Quotient	51	8.535	0.770	0.130	131.000
		Quotient IS	15	3.141	2.600	0.130	8.400
Responder	46	Time_MMR	46	5.174	5.000	0.000	12.000
		Age	46	57.804	58.500	14.000	85.000
		Leukocytes	46	42089.130	11200.000	3400.000	316000.000
		Quotient	46	1.314	0.575	0.016	7.800
		Quotient IS	22	2.225	0.795	0.012	9.200

Group	N Obs	Variable	Std Dev	Coeff of Variation
Nonresponder	51	Time_MMR	16.667	61.000
		Age	16.149	27.675
		Leukocytes	112439.960	123.591
		Quotient	20.937	245.305
		Quotient IS	2.607	82.978
Responder	46	Time_MMR	3.013	58.241
		Age	17.490	30.257
		Leukocytes	64662.715	153.633
		Quotient	1.979	150.600
		Quotient IS	2.630	118.237

Comparison Responder - Nonresponder

The FREQ Procedure

Table of Group by type of *BCR::ABL1* gene fusion

Group<br>Frequency<br>Row Pct	b2a2	b2a2.b3a2	b3a2	e1a2	Total
Nonresponder	21<br>42.00	8<br>16.00	19<br>38.00	2<br>4.00	50
Responder	22<br>47.83	10<br>21.74	14<br>30.43	0<br>0.00	46
Total	43	18	33	2	96

Frequency Missing = 1

Statistics for Table of Group by type of *BCR::ABL1* gene fusion

Statistic	DF	Value	Prob
Chi-Square	3	2.8413	0.4167
Likelihood Ratio Chi-Square	3	3.6123	0.3065
Mantel-Haenszel Chi-Square	1	1.2454	0.2644
Phi Coefficient		0.1720	
Contingency Coefficient		0.1695	
Cramer's V		0.1720	

WARNING: 25% of the cells have expected counts less than 5. Chi-Square may not be a valid test.

Fisher's Exact Test

Table Probability (P)	0.0064
Pr <= P	0.4872

Effective Sample Size = 96
Frequency Missing = 1

Comparison Responder - Nonresponder

The FREQ Procedure

Table of Group by sex

Group / Frequency / Row Pct	sex: F	M	Total
Nonresponder	21<br>41.18	30<br>58.82	51
Responder	19<br>41.30	27<br>58.70	46
Total	40	57	97

Statistics for Table of Group by sex

Statistic	DF	Value	Prob
Chi-Square	1	0.0002	0.9898
Likelihood Ratio Chi-Square	1	0.0002	0.9898
Continuity Adj. Chi-Square	1	0.0000	1.0000
Mantel-Haenszel Chi-Square	1	0.0002	0.9899
Phi Coefficient		-0.0013	
Contingency Coefficient		0.0013	
Cramer's V		-0.0013	

Fisher's Exact Test

Cell (1,1) Frequency (F)	21
Left-sided Pr <= F	0.5765
Right-sided Pr >= F	0.5869
Table Probability (P)	0.1634
Two-sided Pr <= P	1.0000

Sample Size = 97

BCR::ABL1 quotient

The MEANS Procedure

Analysis Variable : Quotient

N Obs	Mean	Median	Minimum	Maximum	Std Dev
97	32.12	25.00	0.02	178.00	32.90

Analysis Variable : Quotient

Group	N Obs	Mean	Median	Minimum	Maximum
Nonresponder	51	35.38	26.00	0.15	178.00
Responder	46	28.50	22.50	0.02	85.00

Analysis Variable : Quotient

Group	N Obs	Std Dev
Nonresponder	51	36.30
Responder	46	28.62

Quotient - U-Test

The NPAR1WAY Procedure

Wilcoxon Scores (Rank Sums) for Variable Quotient
Classified by Variable Group

Group	N	Sum of Scores	Expected Under H0	Std Dev Under H0	Mean Score
Nonresponder	51	2661.0	2499.0	138.406029	52.176471
Responder	46	2092.0	2254.0	138.406029	45.478261

Average scores were used for ties.

Wilcoxon Two-Sample Test

Statistic	2092.0000
Normal Approximation	
Z	-1.1669
One-Sided Pr < Z	0.1216
Two-Sided Pr > |Z|	0.2433
t Approximation	
One-Sided Pr < Z	0.1231
Two-Sided Pr > |Z|	0.2462

Z includes a continuity correction of 0.5.

Kruskal-Wallis Test

Chi-Square	1.3700
DF	1
Pr > Chi-Square	0.2418

Correlation of relative transcript levels of *ESPL1*, *PTTG1* and *PTTG1IP* within the R, NR and control groups (Pearson correlation coefficients).

	**controls** (n=51)	***NR*** (n=51)	***R*** (n=46)
ESPL1/ PTTG1	0.25 (*p*=0.0761)	0.58 (*p*<0.0001)	0.55 (*p*<0.0001)
ESPL1/ PTTG1IP	0.30 (*p*=0.0311)	0.25 (*p*=0.0751)	-0.30 (*p*=0.0444)
PTTG1/ PTTG1IP	0.24 (*p*=0.0940)	0.52 (*p*=0.0001)	0.13 (*p*=0.3947)

Correlation between time until achievement of MMR and clinical parameters in CML patients (Spearman correlation coefficient)

**Parameter**	**Test**	**All (n=97)**	**R (n=51)**	**NR (n=46)**
**Distance**	Spearman	0.10 (*p*=0.334)	-0.22 (*p*=0.150)	0.03 (*p*=0.811)
***BCR::ABL1* gene fusion**	Kruskal-Wallis	0.21 (*p*=0.258)	0.15 (*p*=0.609)	0.31 (*p*=0.182)
**Sex**	U-test	0.04 (*p*=0.665)	0.06 (*p*=0.677)	0.11 (*p*=0.427)
**Age**	Spearman	0.02 (*p*=0.873)	-0.13 (*p*=0.377)	0.20 (*p*=0.153)
**TKI therapy***	U-test	-0.16 (*p*=0.247)	-0.46 (*p*=0.019)	-0.25 (*p*=0.193)

*n=53 (55%), only patients receiving monotherapy with nilotinib or imatinib were included
