# Supplementary material for: Gene Expression Pattern of ESPL1, PTTG1 and PTTG1IP Can Potentially Predict Response to TKI First-Line Treatment of Patients with Newly Diagnosed CML
Source: Cancers (Basel). 2023 May 8;15(9):2652. doi: 10.3390/cancers15092652 (PMC10177117; doi:10.3390/cancers15092652)
Supplement: Supplementary file 1 [file cancers-15-02652-s001.zip › Table S3.pdf]

Distribution of therapies in CML patients (n=96) during the first 12 months after diagnosis

Therapy	Frequency	Percentage
Imatinib	31	32.29%
Nilotinib	22	22.92%
Dasatinib	7	7.29%
Bosutinib	1	1.04%
Imatinib - nilotinib	3	3.13%
Imatinib - dasatinib	2	2.08%
Imatinib - bosutinib	1	1.04%
Nilotinib - dasatinib	2	2.08%
Nilotinib - bosutinib - dasatinib	1	1.04%
IFN - imatinib	1	1.04%
IFN - nilotinib	9	9.38%
IFN - nilotinib - dasatinib	1	1.04%
HU - imatinib	1	1.04%
HU - nilotinib	2	2.08%
HU - dasatinib	1	1.04%
HU - nilotinib - imatinib	2	2.08%
HU - IFN - nilotinib	4	4.17%
Ara-C - dasatinib - nilotinib	1	1.04%
TKI - SZT	4	4.17%
