# Supplementary material for: Gene Expression Pattern of ESPL1, PTTG1 and PTTG1IP Can Potentially Predict Response to TKI First-Line Treatment of Patients with Newly Diagnosed CML
Source: Cancers (Basel). 2023 May 8;15(9):2652. doi: 10.3390/cancers15092652 (PMC10177117; doi:10.3390/cancers15092652)
Supplement: Supplementary file 1 [file cancers-15-02652-s001.zip › Table S4.pdf]

<u>CML patients (NR, R cohorts): rate of *BCR::ABL1* decline, halving and doubling times after 3 months of TKI treatment</u>

No.	***BCR::ABL1*% IS** (ID)**	***BCR::ABL1* % IS** 3M**	**Halving time (days) 3M**	**Doubling time (days) 3M**
NR (n=51)				
NR-1	18	1.579	49	-
NR-2	76	2.000	46	-
NR-3	22	78	-	35
NR-4	100	29	63	-
NR-5	31	0.36	46	-
NR-6	61	6.5	50	-
NR-7	14	1.1	49	-
NR-8	13	2.1	54	-
NR-9	0.48	0.29	114	-
NR-10	0.13	0.19	-	186
NR-11	31*	3.8*	51	-
NR-12	25	14	102	-
NR-13	52	0.19	45	-
NR-14	0.53	0.25	85	-
NR-15	51	19	72	-
NR-16	131	7	48	-
NR-17	52*	19*	71	-
NR-18	33	3.5	50	-
NR-19	43*	35*	242	-
NR-20	4.00*	1.4*	69	-
NR-21	38	31	244	-
NR-22	17	0.54	46	-
NR-23	80	5.1	48	-
NR-24	0.76	2	-	55
NR-25	1.80	3.1	-	125
NR-26	52	2.4	47	-
NR-27	26	14	98	-
NR-28	6.20*	22*	-	35
NR-29	28*	5.8*	57	-
NR-30	12*	11*	541	-
NR-31	8.40*	24*	-	48
NR-32	66	28	78	-
NR-33	3.90*	0.0088*	45	-
NR-34	20*	38*	-	100
NR-35	1.80	1.80	-***	-
NR-36	39	15	73	-
NR-37	77	24	65	-
NR-38	178	3.8	46	-
NR-39	15	0.62	47	-
NR-40	4.1	0.65	54	-
NR-41	30*	18*	113	-
NR-42	2.60*	0.24*	50	-
NR-43	52	9	54	-
NR-44	102	0.8	45	-
NR-45	43	17	74	-
NR-46	57	42	171	-
NR-47	0.92	0.42	82	-
NR-48	45	8.4	55	-
NR-49	20*	20*	-***	-
NR-50	4.20*	39*	-	11
NR-51	7.30	20	-	52
R (n=46)				
R-1	0.01*	0.015*	-	400
R-2	2.50	0.035	46	-
R-3	0.52	0.043	49	-
R-4	0.35	0.032	50	-
R-5	0.50	0	45	-
R-6	30	0	45	-
R-7	5.20	0.077	46	-
R-8	0.55	0.041	48	-
R-9	22	0.085	45	-
R-10	6.00	0.028	45	-
R-11	0.90	0.041	47	-
R-12	0.97	0.018	46	-
R-13	51	0.047	45	-
R-14	1.10	0.0032	45	-

R-15	0.30	0.086	63	-
R-16	68	0.073	45	-
R-17	48	0.061	45	-
R-18	0.18	0.043	60	-
R-19	78	0.088	45	-
R-20	0.12	0.53	-	26
R-21	4.20	2.7	126	-
R-22	34	0.13	45	-
R-23****	3.20	3.1	1455	-
R-24	85	7.2	49	-
R-25	23	0.28	46	-
R-26	73	0.92	46	-
R-27	6.00	0.00	45	-
R-28	36	0.18	45	-
R-29	49	0.042	45	-
R-30	53	1.9	47	-
R-31	55	0.46	45	-
R-32	70	0.18	45	-
R-33	48	0.14	45	-
R-34	9.20	0.0085	45	-
R-35	33	0.26	45	-
R-36	35	0.35	45	-
R-37	79	4.9	48	-
R-38	0.69	0.46	133	-
R-39	40	0.14	45	-
R-40	34	0.15	45	-
R-41	0.18*	0.21*	-	600
R-42	5.60*	0.20*	47	-
R-43	58	17	64	-
R-44	0.67	2	-	45
R-45	81	0.25	45	-
R-46	68	0.71	45	-

CML, chronic myelogenous leukemia; Q, quotient; IS, International Scale; n, number of patients; NR, nonresponder, R, responder.

* Due to non-sufficient sample quality the data of the following sample was used.
** *BCR::ABL1*%Lab values were used in patients with *BCR::ABL1* >10%.
*** Due to lack of slope no halving time or doubling time could be calculated.
**** R Outlier omitted from analysis.
