# Supplementary figures and images for: Gene Expression Pattern of ESPL1, PTTG1 and PTTG1IP Can Potentially Predict Response to TKI First-Line Treatment of Patients with Newly Diagnosed CML
Source: Cancers (Basel). 2023 May 8;15(9):2652. doi: 10.3390/cancers15092652 (PMC10177117; doi:10.3390/cancers15092652)

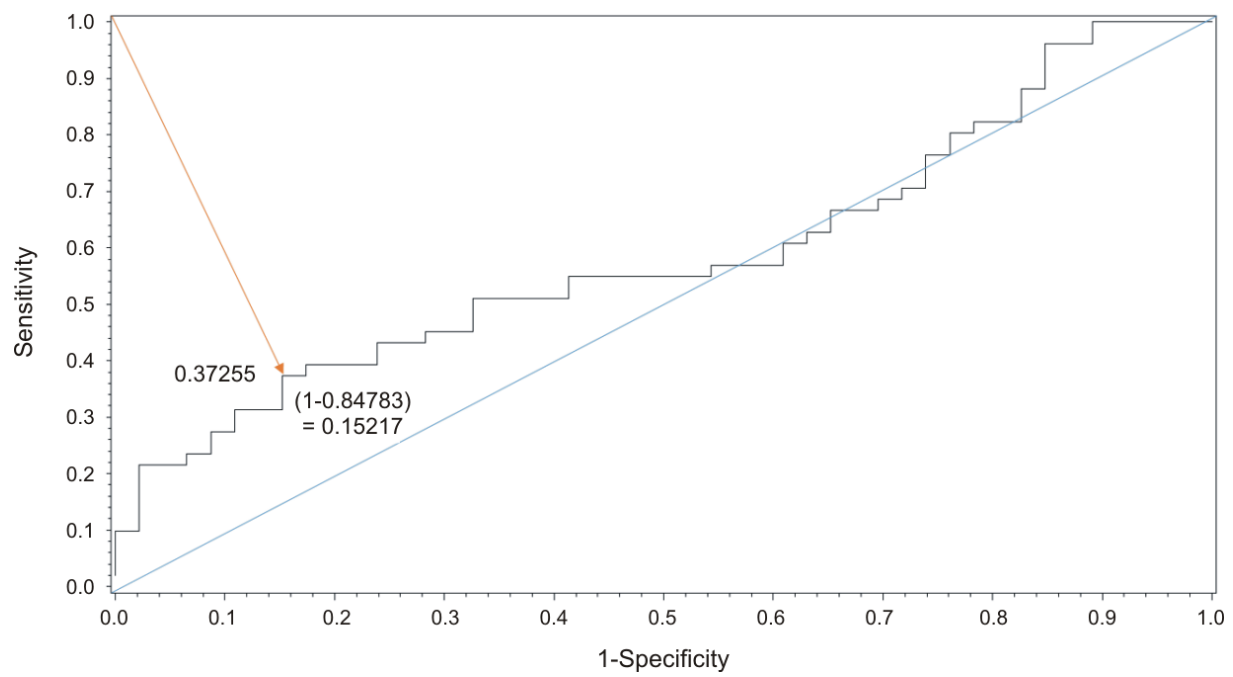

ROC curve for cut-offs of CML samples analyzed (n=97).  
AUC value = 0.587

Supplement: Supplementary file 1 [file cancers-15-02652-s001.zip › Figure S1.pdf]
